# ER-α36-mediated gastric cancer cell proliferation via the c-Src pathway

**DOI:** 10.3892/ol.2013.1416

**Published:** 2013-06-20

**Authors:** XUMING WANG, HAO DENG, FENG ZOU, ZHENQI FU, YING CHEN, ZHAOYI WANG, LIJIANG LIU

**Affiliations:** 1 Department of Pathology and Pathophysiology, School of Basic Medical Science of Wuhan University, P.R. China; 2 Department of Pathology and Pathophysiology, School of Medicine, Jianghan University, Wuhan, Hubei, P.R. China;; 3 Department of Medical Microbiology and Immunology, Creighton University Medical School, Omaha, NE, USA

**Keywords:** gastric cancer, ER-α36, c-src, cyclin D1, proliferation

## Abstract

Previously, a novel variant of estrogen receptor (ER)-α, ER-α36, was identified and cloned and reported to mainly mediate non-genomic estrogen signaling. More recently, we identified that ER-α36 is important for the invasion and lymph node metastasis of human gastric cancer. In the present study, the c-Src signaling pathway was demonstrated to be involved in the non-genomic estrogen signaling mediated by ER-α36 in SGC7901 gastric cancer cells. SGC7901 cells were subjected to the siRNA-mediated knockdown of ER-α36 (PLKO.1-PURO-SP6-ER-α36-L) or transfected with an ER-α36 upregulated expression plasmid (PLJM1-ER-α36-H) and treated with 17β-estradiol (E2β) and PP2, a c-Src protein inhibitor. The expression of ER-α36 and c-src/p-c-Src and cyclin D1 was examined by western blot analysis, and tumor cell growth was analyzed by cell proliferation and nude mouse xenograft assays. The ER variant, ER-α36, was shown to enhance gastric cancer cell proliferation through activation of the membrane-initiated c-Src signaling pathways, indicating that ER-α36 is important for the regulation of proliferation in gastric cancer. In addition, ER-α36 was shown to directly interact with c-Src by immunoprecipitation. The results of the present study indicate that the use of ER-α36 may be a targeted therapeutic approach in gastric cancer.

## Introduction

Gastric cancer is one of the most common forms of cancer worldwide with ∼989,600 new cases and 738,000 mortalities annually, which accounts for ∼8% of new cancers (1). Epidemiological studies of the incidence and prevalence of gastric cancer indicate that the male and female ratio is 2:1 (1–3). The gender difference may not be accounted for by environmental risk factors for gastric cancer, including *Helicobacter pylori* infection, smoking and diet (4–8). The incidence of gastric cancer has been reported to be higher in males than females prior to menopause, however, following menopause, the incidence in females is similar to that of males (9). In patients with prostate cancer, the risk of developing gastric cancer has been identified to be lower in individuals treated with estrogen therapy compared with those who have not received treatment (10–14). In breast cancer, patients treated with anti-estrogen tamoxifen have been identified to exhibit a significantly increased risk of subsequent gastric cancer (15,16). In addition, ovariectomy also significantly increases the risk of gastric cancer in females (17). Taken together, it has been hypothesized that female sex hormones play a protective role against the development of gastric cancer.

It has been well established that the functions of estrogen are mediated by estrogen receptor–α (ER-α) and ER-β (18). ER-α mainly exists in three isoforms, namely ER-α66, ER-α46 and ER-α36 (19). ER-α66 functions as a ligand-dependent transcription factor, regulating gene expression by binding estrogen response elements (EREs) in DNA (18). ER-α46 lacks an AF-1 domain, however, it is able to bind to the ERE and form heterodimers with ER-α66 (20,21). ER-α46 is localized to the plasma membrane, in the cytosol and to the nucleus and mediates rapid estrogen signaling, including activation of the Src/PI3K/AKT pathway (22–24), indicating a possible role of ER-α46 in rapid non-genomic estrogen signaling. ER-α36 differs from ER-α66 in that it lacks the two transcriptional activation domains (AF-1 and AF-2) but retains the DNA-binding, dimerization and the majority of the ligand-binding domains. ER-α36 also mediates rapid estrogen signaling (19). It is a paradox that, on the one hand, estrogen is associated with gastric cancer cells and, on the other hand, the expression of ER-α66 is low and the presence of ER-β in gastric cancer may have a protective effect against the invasiveness of gastric cancer (25,26).

Previously, we reported that ER-α36 protein is expressed in human gastric adenocarcinoma tissues and gastric cancer cell lines, and that ER-α36 expression significantly correlates with tumor invasion and lymph node metastasis in gastric cancer (27). In the present study, the underlying mechanisms by which ER-α36 functions in gastric cancer SGC7901 cells were investigated and the role of the c-src/cyclin D1 pathway was assessed.

## Materials and methods

### Reagents

17β-estradiol (E2β) and PP2 (a Src inhibitor) were obtained from Sigma-Aldrich (St. Louis, MO, USA). Rabbit polyclonal anti-ER-α36 antibody was generated and characterized as described previously (28). Anti-c-Src (sc-19), anti-p-c-Src (sc-81521 and sc-16846-R), anti-cyclin D1 (sc-718) and anti-β-actin (sc-47778) antibodies were purchased from Santa Cruz Biotechnology, Inc. (Santa Cruz, CA, USA). RIPA buffer and the Enhanced BCA Protein Assay kit were from the Beyotime Institute of Biotechnology (Shanghai, China). PVDF membranes were purchased from Millipore (Billerica, MA, USA). Lipofectamine 2000 reagent was from Invitrogen Life Technologies (Carlsbad, CA, USA) and Protein A agarose was from Santa Cruz Biotechnology, Inc. (sc-2001).

### Cell lines

The human gastric cancer cell line, SGC7901, was obtained from the Chinese Academy of Medical Sciences Cell Center of Basic Medicine (Beijing, China). Recombinant cell lines (low and high ER-α36 expression) of gastric cancer SGC7901 cells were generated in the Pathology and Pathophysiology Key Laboratory of Wuhan (China) as described previously (27).

### Cell culture

All cells were maintained in RPMI-1640 medium (Invitrogen Life Technologies) containing 10% fetal bovine serum (FBS) at 37°C in a 5% CO_2_ atmosphere. Prior to treatment with E2β, the cells were changed to phenol-red-free RPMI-1640 medium and 2% FBS for 2–3 days and then maintained in serum-free medium for 6 h prior to experimentation.

### Cell proliferation assay

To examine cell growth in the presence or absence of estrogen, the cells maintained for 3 days in phenol red-free RPMI-1640 medium plus 2% FBS were treated with E2β (0.1 nM) and/or PP2 (10 *μ*M) or ethanol vehicle as a control. Following treatment for 5, 7, 9 and 11 days, the cells were trypsinized and counted with the Scepter™ 2.0 handheld automated cell counter (Merck KGaA, Darmstadt, Germany). Assays were performed in 3 dishes for each time point and all experiments were repeated 3 times.

### Western blot analysis

For the western blot analysis, the cells were washed with cold PBS and lysed in lysis buffer [50 mM Tris-HCl (pH 8.0), 150 mM NaCl, 0.25 mM EDTA (pH 8.0), 0.1% SDS, 1% Triton X-100 and 50 mM NaF] supplemented with protease and phosphatase inhibitors purchased from Sigma-Aldrich. Protein concentrations were determined with the Enhanced BCA Protein Assay kit. The cell lysates were mixed with loading buffer, separated by 12% SDS-PAGE gels and transferred to a PVDF membrane. The membranes were probed with various primary antibodies, appropriate secondary antibodies and visualized with enhanced chemiluminescence detection reagents (DNR Bio-Imaging Systems Ltd., Jerusalem, Israel). The densities of the protein bands were assessed using the TotalLab analysis software (Nonlinear Dynamics Ltd., Durham, NC, USA).

### Nude mouse xenograft assay

Male nude mice (BALB/c nu/nu nude mice, 20–25 g) were purchased from the Hubei Experimental Animal Center (Wuhan, China). All experimental procedures were approved by the Animal Care and Use Committee at the School of Medicine (Wuhan University, Wuhan, China). All experimental procedures were performed in compliance with the National Institutes of Health guidelines on the ethical use of animals. The following cell lines were used: SGC7901, ER-α36 upregulated SGC7901 (High36) and ER-α36-knockdown SGC7901 (Low36) cells. Cells (∼5×10^5^) suspended in PBS were dorsally implanted into nude mice subcutaneously. The tumor volume (V) was measured with a caliper every 4 days and was calculated as V = length × width (cm^2^). After 24 days, all the animals were sacrificed. The tumors were removed and weighed. All tumor tissues were retained for western blot analysis and immunohistochemistry (IHC).

### IHC

Paraffin-embedded tissue sections (5 *μ*m) were dewaxed in xylene and rehydrated in graduated concentrations of ethanol (100, 95, 90, 80 and 70% in PBS, 5 min each solution). Antigen retrieval was performed by incubating the slides with 100 mM sodium citrate solution (pH 6.0) for 20 min. The tumor tissues were stained with antibodies against ER-α36, c-Src and cyclin D1, followed by avidin-biotin-immunoperoxidase visualization. The cell nuclei were stained with hematoxylin. Positively-stained cells were observed using an Olympus microscope (Olympus Corporation, Tokyo, Japan). Immunostained slides were evaluated by two pathologists independently in a blind manner. In the majority of cases, the evaluations of the two pathologists were identical. Any discrepancies were resolved by re-examination and consensus.

### Immunoprecipitation (IP)

E2β (0.1 nM) and/or PP2 (10 *μ*M) were applied for 10 min to stimulate the SGC7901 cells. The cells were washed with cold PBS and lysed with lysis buffer supplemented with protease and phosphatase inhibitors. The protein concentrations were determined using the Enhanced BCA Protein Assay kit. The cell lysates were used for IP. Briefly, the lysates were mixed with antibodies against ER-α36, c-src, p416-c-Src and p527-c-Src in IP buffer [10 g/l HEPES (pH 7.4), 150 g/l NaCl and 0.1% BSA] supplemented with protease inhibitors and were incubated for 1 h at 4°C with gentle agitation. Protein A sepharose beads were added and the samples were incubated for 2 h at 4°C using gentle agitation. Unbound proteins were removed by washing the beads three times in IP buffer. The bound proteins were eluted from the beads with sodium dodecyl sulfate-polyacrylamide gel electrophoresis (SDS-PAGE) sample buffer and the IP samples were analyzed with SDS-PAGE.

### Statistical analysis

The statistical analysis was performed using SPSS 12.0 software (SPSS, Inc., Chicago, IL, USA). Data are presented as the mean ± SD in three replicate samples and compared using the Student’s t-test and analysis of variance. P<0.05 was considered to indicate a statistically significant difference. All experiments were performed at least 3 times to ensure the reproducibility of the results.

## Results

### Estrogen stimulates proliferation of gastric cancer cells via ER-α36

We previously identified that ER-α36 was expressed in a number of gastric cancer cell lines and tissue specimens from gastric cancer patients (27). In connection with these observations, in the present study, the role of ER-α36-mediated estrogen signaling in the proliferation of gastric cancer cells was studied. For this purpose, cell lines were established from the gastric cancer SGC7901 cells that highly expressed recombinant ER-α36 (High36) or exhibited knocked down levels of ER-α36 expression (Low36). Next, E2β (0.1 nM) was used to treat the SCG7901, High36 and Low36 cell lines for various time periods. E2β was demonstrated to promote the proliferation of these cells. High36 had the highest growth rate in response to estrogen treatment and Low36 had the lowest. These results indicate that ER-α36 mediates the estrogen-stimulated proliferation of gastric cancer cells.

### c-Src is involved in ER-α36-mediated mitogenic estrogen signaling in gastric cancer cells

**Figure 1.**
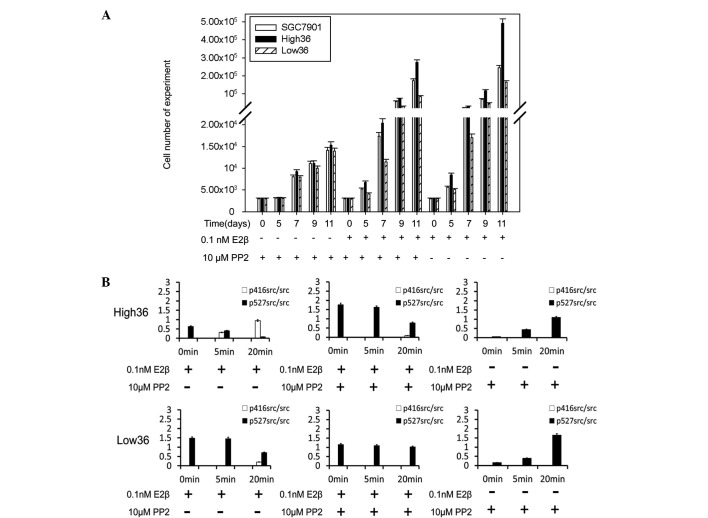
PP2 inhibits the activation of c-Src induced by E2β. (A) PP2 blocks c-Src activation in SGC7901, High36 and Low36 cell lines from days 5–11 and PP2 reduced proliferation in ∼68.91 and 91.56% of High36 and Low36 cells, respectively, at day 11. (B) E2β and/or PP2-stimulated SGC7901, High36 and Low36 cells at 0, 5 and 20 min. Cell lysates were analyzed with anti-p416-c-Src and anti-p527-c-Src antibodies. Anti-c-Src antibody was used to ensure equal loading. Western blot analysis of p-416-c-Src and p-527-c-Src expression in SGC7901, High36 and Low36 cells. E2β, 17β-estradiol.

To observe the mechanisms by which ER-α36 mediates the estrogen-stimulated growth of gastric cancer cells, the c-Src inhibitor, PP2 (10 *μ*M), was used to analyze gastric cancer cell (SGC7901) proliferation. PP2 inhibited the cell proliferation stimulated by E2β in all cell lines (Fig. 1A). PP2 blocked 68.91 and 91.56% of proliferation in the High36 and Low36 cell lines, respectively (Fig. 1A). Western blot analysis using phospho-specific c-Src antibodies revealed that E2β induced phosphorylation of Tyr416 in c-Src and reduced phosphorylation of Tyr527 (Fig. 1B). PP2 inhibited the phosphorylation of Tyr416 induced by E2β and increased the phosphorylation of Tyr527 (Fig. 1B). The phosphorylation of Tyr416 was shown to correlate with the expression levels of ER-α36, indicating that c-Src is involved in the non-genomic estrogen signaling mediated by ER-α36 in gastric cancer cells.

### c-Src is involved in induction of cyclin D1 expression by estrogen in gastric cancer cells

**Figure 2.**
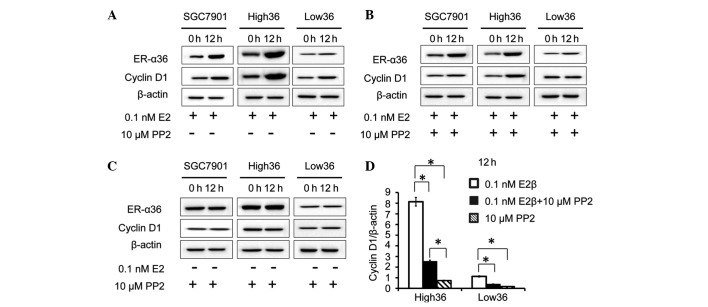
Estrogen induces cyclin D1 expression through activation of the ER-α36 pathway. (A) Western blot analysis of cyclin D1 expression in SGC7901, High36 and Low36 cell lines. Cells were treated with E2β alone. (B) Western blot analysis of cyclin D1 expression in SGC7901, High36 and Low36 cells. Cells were treated with the c-Src inhibitor, PP2 and E2β. (C) Western blot analysis of cyclin D1 expression in SGC7901, High36 and Low36 cells. Cells were treated with the PP2 c-Src inhibitor alone. (D) Estrogen induces cyclin D1 expression through activation of the ER-α36 pathway and the PP2 c-Src inhibitor downregulates cyclin D1 expression in gastric cancer cells (^*^P<0.05, vs. E2β alone). ER, estrogen receptor; E2β, 17β-estradiol.

It is well known that cyclin D1 is an estrogen responsive gene that contributes to the estrogen-stimulated proliferation of breast cancer cells. To examine whether ER-α36-mediated estrogen signaling induces cyclin D1 expression in gastric cancer cells, the cells were treated with E2β (0.1 nM) for 12 h and a western blot analysis was performed to examine cyclin D1 expression. As a result, in the SCG7901 and High36 cell lines, E2β upregulated the expression levels of cyclin D1, whereas in the Low36 cells, E2β failed to induce cyclin D1 expression, indicating that estrogen induces cyclin D1 expression via ER-α36 in gastric cancer cells. Next, the role of c-Src in the induction of cyclin D1 by estrogen was investigated in the gastric cancer cells. The effect of the c-Src inhibitor, PP2, on cyclin D1 induction by E2β was investigated. The cells were treated with E2β and PP2, and a western blot analysis was performed to examine ER-α36 and cyclin D1 expression. PP2 did not alter the ER-α36 expression induced by E2β. The increased levels of cyclin D1 expression induced by E2β were inhibited by PP2, indicating that c-Src iss involved in the induction of cyclin D1 expression induced by E2β in gastric cancer cells (Fig. 2A–D).

### ER-α36 and cyclin D1 are expressed in tumor xenografts

**Figure 3.**
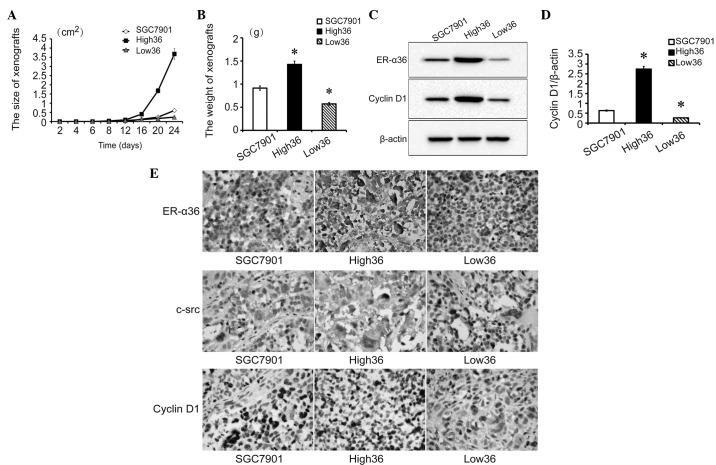
ER-α36 promotes malignant growth of gastric cancer cells in nude mice. (A) The size of the xenografted tumors was examined every 4 days for 24 days. (B) Tumor weight of xenografts at day 24 (^*^P<0.05, vs. SGC7901 control). (C) Western blot analysis of cyclin D1 and ER-α36 expression in nude mice. (D) Western blot analysis of cyclin D1 expression in nude mice (^*^P<0.05, vs. SGC7901 control). (E) Immunohistochemistry (IHC) staining of ER-α36, c-Src and cyclin D1 in the xenografted tumors. ER-α36 and c-Src were observed at the plasma membrane. Cyclin D1 was observed in the nucleus (magnification, ×400). ER, estrogen receptor.

To determine the tumor growth of the cell lines, all cell lines (1×10^6^ cells/nude mice) were transplanted subcutaneously into the skin of the dorsal body of 2 nude mice/cell line. The growth of the transplanted tumors was monitored every 4 days and tumors were detected from day 8. High36 cells formed the largest tumors while Low36 cells formed the smallest tumors (Fig. 3A–B). After 24 days, the nude mice were sacrificed and the tumors were removed. The levels of ER-α36 and cyclin D1 expression in the xenografted tumors were examined with western blot analysis (Fig. 3C and D). Next, the expression of ER-α36, c-Src and cyclin D1 in the xenografted tumors was tested by IHC (Fig. 3E). The expression of ER-α36, c-Src and cyclin D1 was higher in the High36, moderate in the SGC7901 and lower in the Low36 cell lines. In addition, c-Src and cyclin D1 expression was shown to be associated with the expression of ER-α36. These results further indicated that ER-α36-mediated signaling is important for the development of gastric cancer, presumably through c-Src and cyclin D1.

### ER-α36-c-Src interaction is induced by E2β

**Figure 4.**
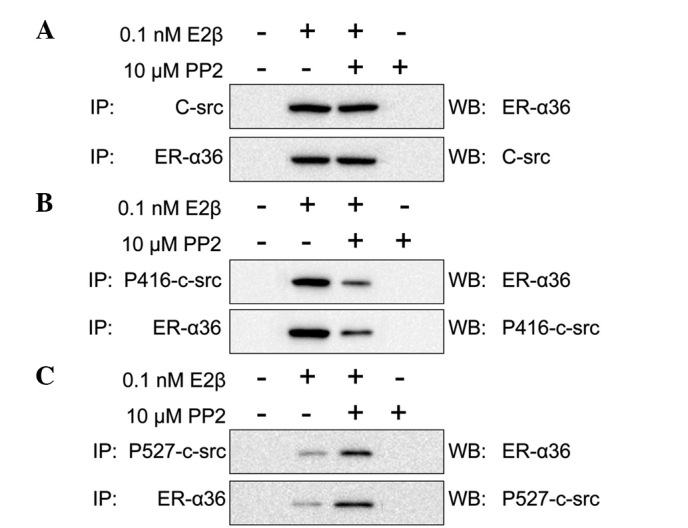
ER-α36-c-Src interaction analysis. Extracts from SGC7901 cells were incubated with E2β and/or with PP2. Formed complexes were pulled down and analyzed using antibodies against (A) c-Src and ER-α36, (B) p416-c-Src and ER-α36 and (C) p527-c-Src and ER-α36. ER, estrogen receptor; E2β, 17β-estradiol.

ER-α36 is known to physically interact with the EGFR/Src/Shc complex and mediate estrogen-induced phosphorylation of epidermal growth factor receptor (EGFR) and c-Src in breast cancer cells (28). Therefore, in the present study, the direct interaction of ER-α36 with c-Src in the SGC7901 cells was analyzed. E2β (0.1 nM) and/or PP2 (10 *μ*M) were used to stimulate the SGC7901 cells for 10 min. Formed complexes were then pulled down and probed using antibodies against ER-α36, c-Src, p416-c-Src and p527-c-Src. When the SGC7901 cells were stimulated by E2β or E2β and PP2 together, ER-α36 was observed to interact with c-Src. However, when the SGC7901 cells were stimulated by PP2 alone, ER-α36 did not interact with c-Src (Fig. 4A). In addition, PP2 decreased the activation of c-Src, which showed a high expression of p527-c-Src and a low expression of p416-c-Src. These observations indicate that p527-c-Src expression is higher than p416-c-Src when SCGC7901 cells are stimulated by E2β and PP2 together (Fig. 4B–C). Therefore, we hypothesized that ER-α36 and c-Src interact in the presence of E2β and that this interaction is not inhibited by PP2. However, PP2 does inhibit the activation of c-Src.

## Discussion

Previous epidemiological studies have reported that the gender difference in the incidence and prevalence of gastric cancer cannot be explained by other factors except estrogen levels. However, a previous study demonstrated that gastric tumor tissues were negative for or showed extremely low levels of ER-α66 (the traditional estrogen receptor) expression (29), generating the query of how the estrogen concentration correlates with the incidence and prevalence of gastric cancer. Our previous study showed that a variant of ER-α, ER-α36, was highly expressed in human gastric tissues and mainly expressed on the plasma membrane and in the cytoplasm of gastric cancer cells. ER-α36 expression was associated with lymph node metastasis, indicating that ER-α36 may be a marker of gastric cancer metastasis (27). Consistent with these observations, in the present study, ER-α36-mediated estrogen signaling was shown to promote the growth of SGC7901 cells *in vitro* and *in vivo*. In addition, ER-α36-mediated estrogen signaling stimulated proliferation of the gastric cancer cells through the activation of the c-Src signaling pathway and the upregulation of cyclin D1 expression.

However, the function of estrogen that stimulated the growth of gastric cancer cells was associated with the concentration of estrogen. Physiologically low concentrations of estrogen (0.1 nM) were identified to promote the growth of gastric cancer cells and the expression of ER-α36. In addition, physiologically high concentrations of estrogen (5 *μ*M) inhibited the growth of gastric cancer cells and the expression of ER-α36 (30). This may explain the male predominance of gastric cancer (9). The pathogenesis of gastric cancer is a multi-step process affected by a number of risk factors. The dysregulation of multiple signaling pathways involved in cell proliferation, invasion and metastasis had been described in gastric cancer (31,32). Results of the current study indicate that ER-α36-mediated estrogen-signaling is important for the development of human gastric cancer.

When we identified that ER-α36 was associated with the incidence and prevalence of gastric cancer, we continued to study the possible downstream signaling mechanisms involved. In a previous study in ER-negative breast cancer cells, c-Src was identified to function as a switch in ER-α36-mediated biphasic estrogen signaling through the EGFR/STAT5 pathway (33). In addition, ER-α36 has been reported to physically interact with the EGFR/Src/Shc complex (28). Consistent with these observations, we hypothesize that c-Src also functions in this manner in ER-α36-positive gastric cancer cells.

c-Src is a non-receptor protein tyrosine kinase that transduces signals involved in a variety of cellular processes, including cell adhesion, invasion, growth and differentiation (34). An important regulatory mechanism of c-Src tyrosine kinase activity involves the control of its phosphorylation status. There are two major phosphorylation sites in the c-Src protein, Tyr416 and Tyr527. When Tyr416 is phosphorylated, it positively regulates c-Src activity and when Tyr416 is dephosphorylated, it negatively regulates c-Src activity (35–37).

By probing the underlying mechanisms of E2β signaling in gastric cancer cells in the present study, 0.1 nM E2β was demonstrated to induce the phosphorylation of c-Src at Tyr416 and the dephosphorylation of c-Src at Tyr527 in all cell lines. These results were more profound in cells with upregulated ER-α36 expression, consistent with the observation that the PP2 c-Src inhibitor inhibited proliferation in these cells. Therefore, the results indicated that the phosphorylation state of c-src-Tyr416 and c-src-Tyr527 functions as a switch to turn on and off non-genomic estrogen signaling depending on the concentration of estrogen.

Cell growth is regulated by proliferation and apoptosis. Cyclin D1 is an important regulatory factor for cell cycle progression and is required to mediate the G_1_ to S transition, in turn leading to DNA synthesis and cell cycle progression (38). The overexpression of cyclin D1 has been documented in a number of carcinomas, including gastric cancer (39–41). A previous study identified a gender difference in MNNG-induced rat gastric carcinogenesis that was hypothesized to be associated with gender differences in cyclin D1/cdk4 expression (42). However, the mechanisms linked to this observation remain unknown. In the present study, E2β induced c-src-Tyr416 phosphorylation in cells with upregulated ER-α36 expression and failed to induce c-src-Tyr527 phosphorylation in cells with knocked down ER-α36 expression. c-Src-Tyr416 phosphorylation increased the levels of cyclin D1 expression and promoted cell proliferation in the upregulated ER-α36 SGC7901 cells, while the opposite occurred in SGC7901 cells with knocked down ER-α36. To further confirm these observations, the expression of ER-α36 and cyclin D1 was analyzed in xenografts of nude mice, which included upregulated ER-α36, knocked down ER-α36 and control SGC7901 cell lines. Cyclin D1 expression was shown to be positively correlated with ER-α36 expression in these xenografts. The results demonstrated that E2β-ER-α36 regulates the phosphorylation of c-src-Tyr-416 and -Tyr-527 to promote the growth of gastric cancer and further indicates that E2β-ER-α36-c-Src is important for proliferation in gastric cancer.

In a previous study, ER-α36 and c-Src were reported to be associated in MDA-MB-231 breast cancer cells (43). In the current study, the interaction between ER-α36 and c-Src was demonstrated in SGC7901 gastric cancer cells. ER-α36 and c-Src were identified to interact in the presence of E2β, and PP2 did not affect this interaction. However, PP2 was observed to inhibit the activation of c-Src. In addition, the association between ER-α36 and cyclin D1 in the SGC7901 gastric cancer cells was induced by E2β.

Since 1983, a number of studies have examined the expression of the ER in gastric cancer (44,45). However, considerable controversy remains with regard to the expression levels of ER and their prognostic value in gastric cancer. Studies have shown that the traditional ER, ER-α66, is absent in gastric cancer (25). The ER has been hypothesized to be associated with gastric cancer, however, to date, no studies have explained the inconsistent negative expression of ER-α66 (25). The identification of the E2-ER α36-c-Src pathway revealed that E2 promotes proliferation in gastric cancer cells by activating ER-α36.

In summary, the results of the present study have demonstrated that ER-α36-mediated estrogen signaling promotes the proliferation of gastric cancer cells, indicating that ER-α36 is important for the development of human gastric cancer. In addition, the study also provides further evidence that c-Src is involved in ER-α36-mediated mitogenic estrogen signaling in gastric cancer cells.
